# Extracellular vesicle fingerprinting: the next generation for cancer diagnosis?

**DOI:** 10.1038/s41392-020-00385-3

**Published:** 2020-11-05

**Authors:** Vanessa Sandim, Robson Q. Monteiro

**Affiliations:** Institute of Medical Biochemistry Leopoldo de Meis, Federal University of Rio de Janeiro, Rio de Janeiro, Brazil

**Keywords:** Tumour biomarkers, Cancer microenvironment

In a recent report, Hoshino and colleagues from several institutions around the world demonstrated that large-scale proteomic analysis of extracellular vesicles (EVs) and particles comprising exomeres and two distinct subpopulations of small and large exosomes (which were named as EVPs) might constitute the next generation for cancer diagnosis.^1^

The authors constructed a robust proteome database of EVPs from human samples (152 control and 274 cancer samples). It included resected normal and malignant tissues, cell lines, and body fluids (blood plasma, blood serum, bone marrow, lymphatic fluid, and bile duct fluid specimens). Besides, authors have also analyzed EVPs of mouse origin (cell lines, tissue explants, and plasma) with essential conclusions when compared to the human database.^1^

EVs are subcellular membrane-containing structures that are released from cells and tissues under both physiological and pathological conditions. EVs have been traditionally classified according to their size and composition, thus comprising a family of heterogeneous structures ranging from exosomes (30–150 nm) to large oncosomes (1–10 μm).^2^ More recently, a novel class of non-membranous nanoparticles (~35 nm), termed exomeres, have been described.^3^

EVs have been implicated in several aspects of cancer biology, including carcinogenesis, tumor growth, drug resistance, and metastatic dissemination.^4^ In this context, there has been significant interest in characterizing their cargo (proteins, nucleic acids, lipids). In addition, it has long been proposed that the detailed analysis of EVs content could support cancer diagnosis, progression, and drug therapy evaluation.^5^

The molecular composition of EVs is a matter of debate, and the search for novel markers is crucial for the refinement of isolation protocols. The proteomic analysis performed by Hoshino and co-workers showed that human EVPs (cell lines, tissues, and most biofluids) display some of the conventional exosome markers, including heat shock cognate 71 kDa protein, heat shock protein HSP 90-beta, CD9, and programmed cell death 6-interacting protein (also known as ALIX). Most remarkably, the authors identified 13 additional proteins in the human samples that deserve further investigation as novel EV markers. On the other hand, many traditional exosome markers were found in <50% frequency in plasma/serum-derived EVPs. Authors suggested that not all pan-exosome markers currently used to characterize these nanovesicles in cell culture translate directly to patient-derived biofluids.

**Fig. 1:**
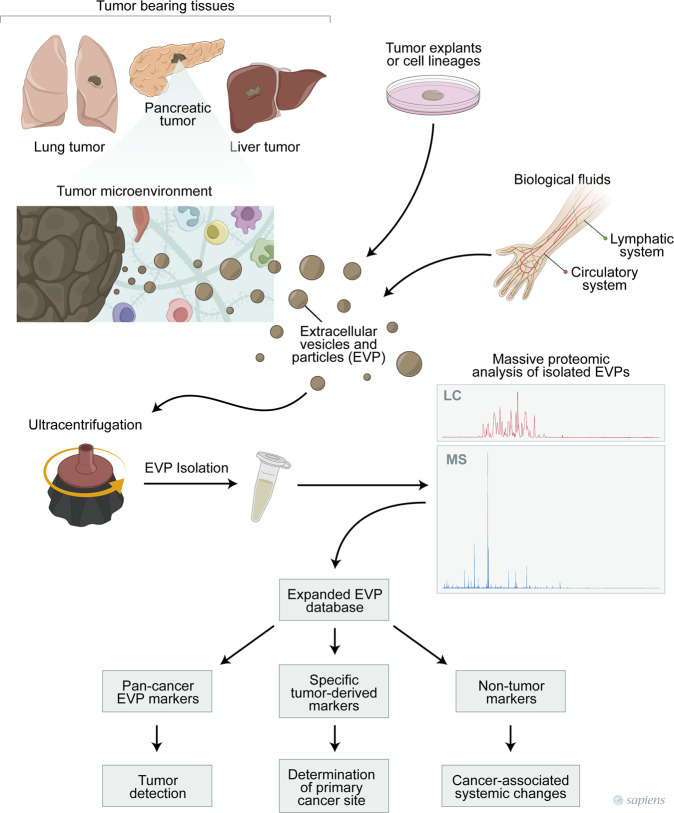
Schematic representation of how EVP proteomic profile may contribute to cancer biology. Isolation of exosomes and exomeres (EVPs) from tissue specimens (such as tumor samples) and/or biological fluids (such as blood and lymph) will require reliable and reproducible methodologies. Further proteomic analysis employing expanded EVP databases may rely on new cancer diagnostic tools and determination of primary tumor sites. In addition, EVP proteomic signatures may potentially help early cancer detection, tumor staging (including the presence of metastatic sites), analysis of tumor microenvironment profiles, and cancer-associated systemic changes

Interestingly, the comparison between the proteome profiling of human and murine-derived EVPs (cell lines and tissue explants) showed high similarity. In contrast, the proteome profiling of plasma-derived EVPs vastly differed between mice and humans. The authors concluded that EVP profiles vary significantly depending on the tissue source and species. Also, the authors ask for caution in studies employing murine plasma-derived EVP proteomes to guide liquid biopsy studies in patients.

Further analysis of EVPs harvested from tumor-tissue, as compared to non-tumor adjacent tissue explants, identified a combination of proteins that distinguished cancer from non-cancer samples. Specific EVP adhesion markers, including CD36, tenascin C, thrombospondin 2, versican, and others, as well as some metabolic enzymes, were suggested as pan-cancer markers. By comparing plasma-derived and tissue-derived EVP proteins, authors were able to discriminate between tumor-derived, adjacent tissue-derived, and distant organ EVPs. Therefore, plasma EVP protein signatures of cancer patients were distinct from those of control subjects and were cancer-type-specific. These findings lead the authors to suggest that the EVP protein profiles could serve as a liquid biopsy tool to detect cancer. Interestingly, several proteins were absent or found at low levels in the explant-derived EVPs but were found exclusively in plasma-derived EVPs from cancer patients. These findings suggest that EVP proteins may reflect cancer-associated systemic changes.

A comparison of the EVP proteomic profiles obtained from samples of distinct cancer types allowed the identification of specific signatures. The determination of the primary site was achieved with either tumor tissue or blood-derived EVPs. Genomic data allow classifying some cancer types into specific subtypes that are biologically distinct and behave differently concerning therapeutic response and clinical outcome. It remains to be determined whether the EVP proteomic profiles may distinguish cancer subtypes with similar complexity as seem with the genomic data.

The tumor microenvironment is a complex tissue comprising tumor cells as well as several different host cells, including leukocytes, endothelial cells, platelets, adipocytes, and others. EVs from both tumor and host cells also constitute the tumor microenvironment, and this reflects the presence of several immune-related proteins in the EVP content. Accordingly, EVP profiling from tumor tissues identified several immune-related proteins. Remarkably, some were exclusively found in the tumor-derived EVPs, such as annexin A3 and some specific integrins. Also, some immune-related proteins were cancer-type specific, possibly reflecting the heterogeneity of tumor microenvironment across cancer types. Several diseases rely on changes in the inflammatory status, including the pre-malignant state. It remains to be determined whether the proteomic profile of EVPs can discriminate between chronic inflammation, pre-malignant, and malignant conditions in the primary sites analyzed in the study. Moreover, additional studies will be necessary to establish reliable EVP signatures to determine cancer staging, in particular those in the early stages of the disease.

Taken together, Hoshino and colleagues established an unprecedented proteomic database of exomeres and exosomes (EVP) from human origin, including cell lines, tumors, and non-tumor explants as well as different biological fluids. The massive analysis allowed the discovery of novel EVP markers and specific signatures across different tumor types (Fig. 1). Most remarkable, authors demonstrated unique proteomic profiles in blood-derived EVPs across distinct cancer types. The establishment of reliable and reproducible EVP isolation procedures, as well as analysis protocols, will be determinant to define the nanovesicle fingerprinting as the next generation for cancer diagnosis.
